# Comparison of Effectiveness between Tension-Free Vaginal Tape (TVT) and Trans-Obturator Tape (TOT) in Patients with Stress Urinary Incontinence and Intrinsic Sphincter Deficiency

**DOI:** 10.1371/journal.pone.0156306

**Published:** 2016-05-26

**Authors:** Hyeong Gon Kim, Hyoung Keun Park, Sung Hyun Paick, Woo Suk Choi

**Affiliations:** Department of Urology, Konkuk University School of Medicine, Konkuk University Medical Center, Seoul, Korea; Northwestern University Feinberg School of Medicine, UNITED STATES

## Abstract

**Background:**

The aim of this study was to compare the two types of mid-urethral slings for stress urinary incontinence (SUI) with intrinsic sphincter deficiency (ISD).

**Methods:**

This retrospective study included patients who underwent tension-free vaginal tape (TVT) procedure or transobturator tape (TOT) procedure by a single surgeon for SUI with ISD, defined as Valsalva leak point pressure (VLPP) < 60 cmH_2_O in a urodynamic study. Cases of neurogenic bladder, previous SUI surgery, and concomitant cystocele repair were excluded. The primary outcome was treatment success at 12 months, defined by self-reported absence of symptoms, no leakage episodes recorded, and no retreatment.

**Results:**

Among the 157 women who were included in the final analysis, 105 patients received TVT and 52 patients received TOT. Age, underlying diseases, Stamey grade, cystocele grade, and presence of urge incontinence were not significantly different between the two groups. Urodynamic parameters including maximal urethral closing pressure, detrusor overactivity, VLPP, urethral hypermobility (Q-tip ≥ 30°), were also comparable between the two groups. Success rate was significantly higher in the TVT group than in the TOT group (95.2% vs. 82.7%, p = 0.009). On multivariate analysis, only TOT surgery (OR = 3.922, 95%CI = 1.223–12.582, p = 0.022) was a risk factor for failure following surgical treatment.

**Conclusion:**

TVT is more effective than TOT in treatment of female SUI with ISD.

## Introduction

Currently, the main surgical treatment option for SUI is a mid-urethral sling via the retropubic or transobturator approach [1]. Although tension-free vaginal tape (TVT) procedure via the retropubic approach is considered as the gold standard with an effective long term result, recently, the transobturator tape (TOT) procedure via the transobturator approach has been performed more frequently due to low rates of complications including bladder or bowel injuries [2, 3]. Several randomized controlled trials have reported that the efficacies of the two approach techniques are comparable [4–6]. One large randomized controlled study showed a similar success rate between TVT (80.8%) and TOT (77.7%) at the 1 year follow up [4]. A recent meta-analysis of TVT and TOT also showed that TVT and TOT had similar subjective and objective cure rates [7].

However, TVT procedure is preferred in some of the more complicated cases such as in patients with high symptom severity grade, a history of failure of previous surgical treatment, and especially, intrinsic sphincter deficiency (ISD) [8]. Especially, in patients with ISD, TVT was performed more frequently and it showed a complete cure rate of 74–86% in long-term follow up studies [9–12]. On the other hand, due to the recent widespread use of TOT procedure, several studies reported a similar success rate of TOT in ISD patients, when compared to that of TVT [13–15]. One study showed a success rate of more than 90% at the 2-year follow up [13].

However, there are few studies comparing TVT and TOT for SUI with ISD, and these studies have shown conflicting results [15–18]. One study showed comparable success rates between the two surgical approaches, and a lower complication rate in the TOT group [16]. On the other hand, other studies reported that TVT is superior to TOT in the treatment of SUI with ISD [17, 18]. These controversial results might have resulted from the diversity among surgeons. In each previous study, surgeries were performed by several surgeons [15–18].

The aim of this study was to compare the success rate of TVT and TOT performed by a single surgeon for SUI with ISD and to identify the prognostic factors for success of the treatments.

## Materials and Methods

### Patients and Subgroups

This retrospective study was approved by the Institutional Review Board of Konkuk University Medical Center.

A total of 927 females underwent mid-urethral sling surgery for female urinary incontinence by a single surgeon between August 2005 and December 2013. After exclusion of the other types of mid-urethral sling surgeries (e.g. mini-TOT and TVT-O), 869 (93.7%) patients underwent TOT or TVT. In addition, 483 patients were excluded because of follow up duration less than 1 year (n = 389), lack of urodynamic profiles (n = 55), history of previous anti-incontinence surgery (n = 15), concomitant cystocele repair (n = 10), neurologic disease (n = 11), neurogenic bladder (n = 3) and pelvic surgeries other than hysterectomy (n = 10).

Among the remaining 386 patients, 157 (40.7%) patients were identified as SUI patients with ISD and were included in the final analysis. The patients with ISD were divided into the TVT group (n = 105, 67%) and the TOT group (n = 52, 33%).

### Preoperative Evaluation

Preoperative evaluation included history taking, urinalysis, urine culture, urogynecologic examination, Q-tip test and urodynamic study. Severity of SUI was graded as Stamey incontinence score (grade 0, continent; grade 1, loss of urine with sudden increase in abdominal pressure such as from coughing, sneezing, or laughing; grade 2, leaks with lesser degrees of physical stress such as walking, standing erect from a sitting position, or sitting up in bed; grade 3, total incontinence, urine is lost without any relation to physical activity or position) [19]. Urogynecologic examination was performed and pelvic organ prolapse was evaluated with the Pelvic Organ Prolapse Quantification (POPQ) system [20, 21]. Urethral hypermobility was defined as mobility ≥ 30° by the Q-tip test [22].

Urodynamic study included noninvasive uroflowmetry, urethral pressure profilometry, filling cystometry with VLPP followed by pressure flow study according to the International Continence Society’s Good Urodynamic Practice and Standardized Terminology guidelines [20, 23]. All urodynamic examinations were performed using Medtronic Duet Logic Urodynamic System (Medtronic Inc., Minneapolis, MN, USA) and a water-filled 2-lumen catheter (Mediwatch UK Ltd., Rugby, UK).

The method of VLPP measurement was similar to the previously reported standard technique [24], and it was identical throughout the entire period of time. The external pressure transducers were zeroed to atmospheric pressure and the reference height of the transducers was defined as the upper edge of the symphysis pubis. After bladder filling with 200 ml normal saline, VLPP measures were attempted. Valsalva maneuvers were repeated more than 10 times. VLPP was reported as the minimal vesical pressure at which leakage was observed. ISD was defined as Valsalva leak point pressure (VLPP) of less than 60 cmH2O [23].

### Surgical Procedures

No definite criteria were used for the selection of slings. The type of sling was selected based on the patient’s preference after detailed information of both surgeries was provided, or based on the surgeon’s preference when the patient could not decide regarding the type of surgery. The TVT operation was performed as described by Ulmsten [25]. The only difference from the Ulmsten’s procedure was that it was performed under general anesthesia. Therefore, the cough-stress test was not performed in this study. Tension of the tape was adjusted by positioning the tape lying loosely under the midurethral area and by confirming the passage of Metzenbaum scissors freely between the urethra and the mesh. The TOT operation was performed in the outside-in manner as described by Delorme [26]. TOT was also performed under general anesthesia, and the cough-stress test was not performed. The tension adjustment method was identical to the method used in TVT operation.

### Comparison of Postoperative Outcome

Postoperative evaluation was performed routinely at 6 months and 12 months after the treatment. Success was defined as self-reported absence of urine leakage under any circumstance, no leakage episodes recorded, and no retreatment. All other cases except for successful cases were regarded as failure. Patients were also divided into the success group and the failure group according to the incontinence status. Success rate at 12 months was compared between the TVT and TOT groups. Various clinical and urodynamic variables were compared between the success group and the failure group to identify the prognostic factors of success in all patients.

### Statistical Analysis

Statistical analysis was performed with commercial statistical software SPSS ver. 19.0 (SPSS Inc., Chicago, IL, USA). All patient records were anonymized and de-identified prior to analysis. Student’s t-test and chi-square test were used to compare characteristics and success rate between the two groups. Logistic regression analysis was used to determine the factors related to success of treatment.

## Results

Mean age of patients in the TVT group was 53.6 ± 10.1 years, and mean age of patients in the TOT group was 54.6 ± 9.9 years (p = 0.574). Baseline characteristics including body mass index, underlying disease, Stamey symptom grade, proportion of mixed incontinence were not significantly different between the two groups (Table 1). Comparison of urodynamic parameters also showed that there was no difference in the maximum flow rate, post-void residual urine volume, maximal urethral closing pressure (MUCP), VLPP and the frequency of detrusor overactivity. Also, the frequency of urethral hypermobility (Q-tip ≥ 30°) and weight of the pad as assessed by the pad test were not significantly different between the two groups.

**Table 1:** Comparison of baseline characteristics between the TVT and TOT groups.

	TVT group (n = 105)	TOT group (n = 52)	P-value
Age (y)	53.6 ± 10.1	54.6 ± 9.9	0.574
Height (cm)	155.3 ± 4.9	155.3 ± 5.6	0.971
Weight (kg)	59.8 ± 8.8	60.1 ± 7.6	0.796
Body mass index (kg/m^2^)	24.8 ± 3.5	25.0 ± 3.4	0.701
Hypertension (n, %)	32 (30.5%)	12 (23.1%)	0.331
Diabetes (n, %)	14 (13.3%)	4 (7.7%)	0.296
Previous Hysterectomy (n, %)	9 (8.6%)	5 (9.6%)	0.829
Cystocele grade (n, %)			0.692
Grade 0	12 (11.4%)	5 (9.6%)	
Grade 1	55 (52.4%)	31 (59.6%)	
Grade 2	38 (36.2%)	16 (30.8%)	
POPQ, Aa (cm)	-1.4 ± 0.9	-1.5 ± 0.8	0.267
POPQ, Ba (cm)	-1.9 ± 1.0	-1.9 ± 0.9	0.825
Urgency (n, %)	64 (61%)	34 (65.4%)	0.589
Urge incontinence (n, %)	45 (42.9%)	29 (55.8%)	0.127
Stamey Grade (n, %)			0.552
Grade I	3 (2.8%)	1 (1.9%)	
Grade II	51 (48.6%)	21 (40.4%)	
Grade III	51 (48.6%)	30 (57.7%)	
Weight of the pad (g)	50.6 ± 45.4	57.2 ± 80.4	0.585
Maximal flow rate (ml/sec)	22.9 ± 9.0	25.8 ± 10.9	0.106
Post-void residual urine (ml)	15.9 ± 26.7	25.9 ± 24.0	0.998
MUCP (n, %)	55.4 ± 22.7	55.9 ± 25.0	0.907
< 40 cmH_2_O	30 (28.6%)	16 (30.8%)	0.776
≥ 40 cmH_2_O	75 (71.4%)	36 (69.2%)	
Detrusor overactivity (n, %)	9 (8.6%)	7 (13.5%)	0.340
VLPP (cmH_2_O)	46.9 ± 9.6	44.8 ± 9.9	0.200
Urethral hypermobility (n, %)	85 (81.0%)	45 (86.5%)	0.383

Values are presented as mean ± standard deviation or the number of patients (%).

TVT, tension-free vaginal tape; TOT, trans-obturator tape; POPQ, Pelvic Organ Prolapse Quantification; MUCP, maximum urethral closing pressure; VLPP, Valsalva leak point pressure

Success rate at 12 months was significantly higher in the TVT group than in the TOT group. Twelve months after the operations, the cumulative cure rates in the TVT group and the TOT group were 95.2% and 82.7%, respectively (p = 0.009).

Univariate analysis to identify the factors affecting failure after treatment showed that TOT type of surgery (OR = 4.186, 95% CI = 1.325–13.222, p = 0.015), and Stamey symptom grade 3 (OR = 3.824, 95% CI = 1.023–14.286, p = 0.046) were related to failure at 12 months after surgeries. On multivariate analysis, TOT type of surgery was the only risk factor that was related to failure following surgical treatment (OR = 3.922, 95%CI = 1.223–12.582, p = 0.022) (Table 2).

**Table 2:** Multivariate analysis of factors related to treatment failure.

Parameters	Univariate			Multivariate		
	OR	95% CI	*p* value	OR	95% CI	*p* value
Age (y)	1.035	0.980–1.093	0.213			
Obesity (BMI ≥ 25 kg/m^2^)	0.686	0.219–2.148	0.517			
Hypertension	2.072	0.675–6.362	0.203			
Diabetes	0.570	0.070–4.637	0.599			
Cystocele Grade			0.745			
Grade 0	ref	ref	ref			
Grade I	1.870	0.221–15.816	0.566			
Grade II	1.280	0.133–12.297	0.831			
Urgency	3.977	0.858–18.437	0.078			
Urge incontinence	3.086	0.924–10.302	0.067			
Stamey Grade (III vs. I-II)	3.824	1.023–14.286	0.046	3.543	0.931–13.491	0.064
Weight of the pad (g)	1.004	0.997–1.011	0.230			
Urethral hypermobility	0.346	0.043–2.765	0.317			
Maximal flow rate (ml/sec)	1.019	0.966–1.075	0.480			
Post-void residual urine (ml)	0.985	0.949–1.022	0.433			
Low MUCP (< 40 cmH_2_O)	1.931	0.630–5.917	0.249			
Detrusor overactivity	1.536	0.312–7.571	0.598			
TOT Operation (vs. TVT)	4.186	1.325–13.222	0.015	3.922	1.223–12.582	0.022

BMI, body mass index; MUCP, maximum urethral closing pressure; TOT, trans-obturator tape; TVT, tension-free vaginal tape

## Discussion

TVT has been accepted as a standard treatment for SUI, based on numerous evidences of reporting the long-term efficacy [27]. However, considering the risk of bladder injury and bleeding during the TVT procedure, TOT was developed as a more safe method than TVT [2].

Several randomized controlled studies showed that the efficacy of TOT was comparable to that of TVT [4–6]. One randomized controlled study that compared the safety and efficacy of TOT to TVT concluded that TOT is not inferior to TVT in the treatment of stress urinary incontinence and TOT results in fewer bladder perforations [5]. Also, another study reported that the overall objective cure rate was 71.4% for TVT and 77.3% for TOT, without a statistical difference at a mean follow-up of 31 months [6].

However, TVT was preferred over TOT for patients with ISD, because TVT is believed to be a more obstructive technique than TOT [8]. Although there are a few concerns about the low cure rate, currently, the use of TOT in ISD patients is increasing and it was reported to be effective [13–15]. However, there are few studies that reported comparative results between TVT and TOT in patients with ISD [15–18]. A retrospective study which compared the efficacy of SPARC^®^ (TVT) and MONARC^®^ (TOT) in patients with ISD showed that the success rate of each surgery was not different (76% in SPARC vs. 77% in MONARC, p > 0.05) [16]. On the contrary, another study that compared pubovaginal sling, TVT and TOT in patients with ISD reported that the complication rate was similar across the surgical methods, but the cure rate was lower in the TOT group (34.9%) than in the PVS group (87.3%) or the TVT group (87.0%) [17]. In that study, the failure rate of TOT was 4.6 fold higher than that of other techniques. A recent study also reported that TOT was nearly 6 times more likely to fail than TVT at 3 months after surgery in patients with borderline maximum urethral closure pressure (≤42 cm H_2_O) [18].

Our study also showed similar results for preferring TVT. The TVT group showed a significantly higher success rate than the TOT group (95.2% vs. 82.7%, p = 0.009). On multivariate analysis, type of surgery was the only risk factor for failure following surgical treatment and the risk of treatment failure in patients who underwent TOT was about 4.0 times higher than that in patients who underwent TVT.

There are no definite evidences that explain why TVT had a superior result than TOT in patients with ISD. As a theoretical explanation, tension vector might have affected the cure rates. One anatomic study showed that tension vectors of TVT are more vertical from the urethra, and tension vectors of TOT are more horizontal from the urethra [28]. In addition, a more proximal location of TOTs might be related to the failure following surgery. One study showed that TOTs are located more proximally than TVTs, using translabial 3D ultrasound [29]. It is well known that sling migration to the bladder neck or the proximal urethra is related to recurrence following surgery [30]. As another explanation, one study showed that surgical outcomes of TVT and TOT were influenced differently by other conditions [31]. The authors investigated the factors related to the cure rate, and they showed that the effectiveness of TVT was influenced by only detrusor overactivity, but the outcome of TOT was influenced by both detrusor overactivity and MUCP. This implies that TVT is a more obstructive surgery that could support the pelvic floor muscles regardless of MUCP, even in cases with severe impairment.

In this study, both TVT and TOT were performed contemporarily by a single experienced surgeon. This is the strength of our study because it increases the objective comparability. Some surgeons might adjust the tension of the mid-urethral sling according to the preoperative VLPP results, especially in patients with low VLPP. Furthermore, the degree of tension adjustment according to VLPP might differ depending on the surgeon. The uneven tension case by case and surgeon variation increase the risk of bias when comparing outcomes, and lead to confusing results. For example, several studies reported that VLPP did not affect the surgical outcome of TOT or TVT [32, 33]. These results might have been affected by the physician’s variation in adjusting the tension.

Therefore, previous conflicting results of TVT and TOT comparative studies might also have been affected by the risk of same bias. None of the previous studies used single surgeon data. Our study is meaningful because similar tension adjustment according to VLPP was performed by a single surgeon in both types of surgeries.

Stamey grade was an independent factor that was related to treatment failure in this study. The importance of Stamey grade in SUI surgery was well demonstrated in previous studies [34–36]. Stamey grade was found to have a positive correlation with VLPP [34]. In addition, a high Stamey symptom grade was an independent clinical factor that predicted the presence of ISD [35]. Furthermore, Ryu et al. also reported that Stamey grade rather than VLPP was important for predicting subjective quality of life and improved incontinence-related quality of life after inside-out transobturator midurethral sling [36].

This study had several limitations. First of all, this study was not a randomized controlled study, but a retrospective study. However, baseline characteristics including VLPP and MUCP were not statistically different between the two groups. Second, the number of patients in the TOT group was lower than that in the TVT group. This was also one of the limitations of a retrospective study. Because previous evidences suggested that TVT is superior in patients with ISD than TOT, TVT was performed more frequently in these patients. Third, a blind review of urodynamic findings was not performed in this study. However, all urodynamic studies were performed by a single physician, and the findings were reported by that physician. Fourth, a relatively short follow-up duration is another limitation of this study. A comparison of long-term results for more than 3 years will be performed in our subsequent study. Fifth, we did not include data about complications of surgeries. Because the aim of this study was to compare the efficacy between the surgeries, only limited data on outcomes were collected. Lastly, the success rate of TOT was relatively high, when compared to that in a previous study which reported a success rate of TOT of 75% in patients with ISD. This might be due to the short follow up duration and evaluation of success by only the subjective method without objective cure. In spite of these limitations, we think that this study is meaningful because it showed that the efficacy of TVT is superior to that of TOT in ISD patients.

## Conclusions

In conclusion, this study showed that TVT is more effective than TOT in treating SUI patients with ISD, although TOT also showed a good surgical outcome. These results suggest that a better surgical outcome can be achieved via the retropubic approach than via the transobturator approach in patients with ISD. For a more definite conclusion, further study in a larger population and a randomized controlled trial are required.

## Supporting Information

S1 File The entire dataset composed of the preoperative parameters and the surgical results. (XLSX)
